# Draft Genome Sequence of a *Pseudomonas aeruginosa* NA04 Bacterium Isolated from an Entomopathogenic Nematode

**DOI:** 10.1128/genomeA.00746-17

**Published:** 2017-09-07

**Authors:** Rosalba Salgado-Morales, Nancy Rivera-Gómez, Luis Fernando Lozano-Aguirre Beltrán, Armando Hernández-Mendoza, Edgar Dantán-González

**Affiliations:** a Laboratorio de Estudios Ecogenómicos, Centro de Investigación en Biotecnología, Universidad Autónoma del Estado de Morelos, Cuernavaca, Morelos, Mexico; b Programa de Genómica Evolutiva, Centro de Ciencias Genómicas, Universidad Nacional Autónoma de México, Cuernavaca, Morelos, Mexico; c Centro de Investigación en Dinámica Celular, Universidad Autónoma del Estado de Morelos, Cuernavaca, Morelos, Mexico

## Abstract

We report the draft genome sequence of Gram-negative bacterium *Pseudomonas aeruginosa* NA04, isolated from the entomopathogenic nematode *Heterorhabditis indica* MOR03. The draft genome consists of 54 contigs, a length of 6.37 Mb, and a G+C content 66.49%.

## GENOME ANNOUNCEMENT

The gammaproteobacterium *Pseudomonas aeruginosa* is Gram negative, aerobic, and highly versatile. It is distributed in different ecological niches, including water and soil, and in association with host organisms (1). The genus *Pseudomonas* is particularly interesting because of its importance as an opportunistic pathogen in humans and its biotechnological potential (2, 3).

We isolated a bacterium from the entomopathogenic nematode *Heterorhabditis indica* MOR03, recovered from soil samples cultivated with sugarcane in Yautepec, Morelos, Mexico (18°55′16.9″N and 99°02′24.0″W’). A previous analysis of 16S rRNA gene sequences showed that the bacterium has a 100% sequence identity with *Pseudomonas aeruginosa*. In addition, this strain shows potential for the biological control of pests and is interesting as a model for studying the interactions between bacteria and host nematodes.

We obtained the genome sequence of *Pseudomonas aeruginosa* NA04. The strain was grown in Luria broth (LB) and incubated for 12 h at 30°C with shaking at 250 rpm. Genomic DNA was extracted with the ZR fungal/bacterial miniprep kit (Zymo Research), and 5 µg of genomic DNA was sequenced using the Illumina HiSeq platform (2 × 300 bp paired-end) approach. We obtained a total of 9,447,636 reads that were quality trimmed and error corrected using DynamicTrim (SolexaQA++) Perl script (4). Reads were assembled using SPAdes version 3.5.0 (5). The draft genome consists of 54 contigs with a total length of 6,375,895 bp. The largest contig was 891,545 bp, the *N*_50_ value was 757,654 bp, and *L*_50_ was reached in 4 contigs. The genome sequence of *P. aeruginosa* PAO1 was used as a reference. The total percentage of aligned bases in the reference is 96.08%, which was used to order the contigs in Mauve (6). Automated annotation was made with Rapid Annotations using Subsystems Technology (RAST) (7). *P. aeruginosa* NA04 has a G+C content of 66.49% and includes 5,823 coding sequences. RNAmmer and ARAGORN obtained 7 rRNAs (5S, 16S, 23S) and 67 tRNAs (8, 9). The analysis of the concatenated sequences (*gyrB*,16S, 23S of rRNA genes) of *P. aeruginosa* NA04 shows that it is closely related to *P. aeruginosa* M18 (NC_017548), isolated from the rhizosphere soil of sweet melon (10). The genome of *P. aeruginosa* NA04 includes genes that code for chitinase and a chitin binding protein. Because the exoskeletons of the insects are constituted with chitin, these could influence the insecticide activity. Also, the genome contains the gene coding for a potent toxin (exotoxin A) that affects the protein synthesis of the host cells and genes encoding the RTX-like toxins found in the entomopathogenic bacterium *Photorhabdus luminescens* (11). These genes are necessary for the production of siderophores involved in the efficient uptake of iron, an important factor for the colonization of the host (12).

### Accession number(s).

This whole-genome shotgun project has been deposited at DDBJ/ENA/GenBank under the accession no. MYFK00000000. The version described in this paper is version MYFK01000000.
